# MLB1 Astrovirus in Children with Gastroenteritis, Italy

**DOI:** 10.3201/eid2001.131259

**Published:** 2014-01

**Authors:** Maria Cristina Medici, Fabio Tummolo, Adriana Calderaro, Gabriella Elia, Krisztiàn Banyai, Flora De Conto, Maria Cristina Arcangeletti, Carlo Chezzi, Canio Buonavoglia, Vito Martella

**Affiliations:** Università degli Studi di Parma, Dipartimento di Medicina Clinica e Sperimentale, Parma, Italy (M.C. Medici, F. Tummolo, A. Calderaro, F. De Conto, M.C. Arcangeletti, C. Chezzi);; Università Aldo Moro di Bari, Dipartimento di Medicina Veterinaria, Valenzano, Italy (G. Elia, C. Buonavoglia, V. Martella);; Centre for Agricultural Research, Hungarian Academy of Sciences, Budapest, Hungary (K. Banyai)

**Keywords:** astrovirus, viruses, MLB1, children, gastroenteritis, Italy, enteric infections

**To the Editor:** Astroviruses are notable agents of gastroenteritis in many mammalian and avian hosts. Astroviruses are nonenveloped RNA small, round, viruses (SRVs) with a single-stranded, positive sense RNA of 6.1 to 7.9 kb (*1*). The genome contains 2 nonstructural genes, open reading frame (ORF) 1a and 1b, and a capsid gene, ORF2, with short 5′ and 3′ untranslated regions. Human astroviruses, a major cause of gastroenteritis, are classified in the human astrovirus species, comprising 8 serotypes (*1*). Recently, astroviruses genetically unrelated to canonical human astroviruses have been identified in human stools in several countries. These unusual astroviruses form 2 main genetic clades. One clade contains MLB1, MLB2, and MLB3 (*2**–**4*). The second clade contains VA1, VA2, VA3 (also known as HMO-C, HMO-A, and HMO-B, respectively) and VA4 (*5**,**6*). More recently, a VA1/HMO-C–like virus was detected in brain tissue from an immunocompromised child with encephalitis (*7*). The discoveries of these viruses provide novel candidate agents of human disease and raise concerns inherent of possible zoonotic implications. Here we describe the detection and genome characterization of MLB1-like astrovirus in a 4-year-old male child hospitalized with severe gastroenteritis during January 2007 at the University Hospital of Parma, Italy. Clinical signs included vomiting and severe diarrhea, with moderate dehydration. The child was treated with rehydration and maintenance therapy (balanced glucose-electrolyte solutions) and completely recovered after 3 days. 

Fecal samples collected at admission were subjected to routine virologic (electron microscopy [EM], cell cultures, latex agglutination, and reverse transcription PCR) and bacteriologic (culturing with selective and differential media) examinations. Fecal samples tested negative for common bacterial (*Clostridium difficile, Shigella* spp., *Salmonella* spp., *Yersinia enterocolitica, Staphylococcus aureus,* and *Campylobacter* spp.) and viral (adenovirus, rotavirus, norovirus, human astrovirus, enterovirus and sapovirus) enteric pathogens. However, through EM, SRV particles were observed in the feces of the patient (Figure, panel A). Despite several efforts with additional consensus primer sets for calicivirus and enterovirus, it was not possible to identify the SRVs detected by EM, and the case was archived as undiagnosed. However, beginning in 2008, several astroviruses genetically unrelated to canonical human astroviruses have been described (*2*). Broadly reactive consensus primers for astrovirus (*8*) spanning the RNA-dependent RNA polymerase (RdRp, ORF1b), along with sets of specific primers for these novel astroviruses (*2*), have been designed. By using these sets of primers, astrovirus RNA was detected in the sample. Upon sequence analysis of a small ORF2 fragment, the astrovirus strain (ITA/2007/PR326) displayed up to 97.8% nucleotide identity to MLB1-like viruses. Fragments of the genomic RNA in ORF1a (1,173 nt), ORF1b, and the genome 3′ end (2,930 nt), including the full-length ORF2, were sequenced by using primers specific for MLB1-like astroviruses and RACE 3′ protocols; total genome coverage was 66.5% (4,103/6,169 nt). In all the genome regions sequenced, the virus displayed the highest sequence identity to MLB1-like astroviruses (97.4%–98.2%). The partial ORF1b, full-length ORF2, and 3′ UTR sequence was submitted to GenBank under accession no. KF417713.

**Figure:**
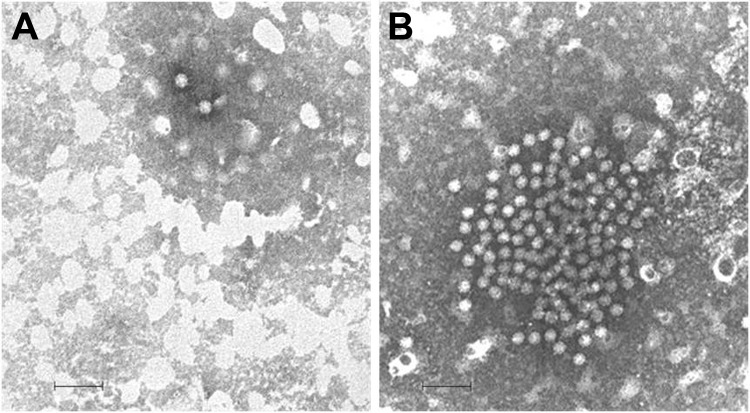
Electron microscopy images of astrovirus-like particles in fecal samples from 2 patients in Italy: A) strain ITA/2007/PR326, from a 4-year-old child hospitalized in January 2007; and B) strain ITA/2008/PR3147, from a 14-month-old child hospitalized in November 2008. The viral particles are ≈28–30 nm in diameter. Scale bars indicate 100 nm.

We hypothesized that the MLB1-like astrovirus was the causative agent of the acute gastroenteric disease observed in the child, because this virus was the only infectious agent identified in samples from this patient. The virus was also present in fecal samples at high titers, detectable by using EM, without using immune-precipitating sera. However, in a case–control study for MLB1-like astroviruses in India, no clear association was established between MLB1-like viruses and diarrhea, and no differences were observed in the viral load between symptomatic and asymptomatic subjects (*9*). We cannot rule out the presence of other, yet unrecognized, viruses in the sample, because we did not perform massive sequencing.

To assess whether the reported case was sporadic or if other infections by MLB1-like astroviruses occurred, we screened fecal samples of 3 other patients hospitalized with gastroenteritis at University Hospital of Parma during 2007–2010 that were negative for common bacterial and viral enteric pathogens and that contained SRVs upon EM observation. In this screening, an additional MLB1-like astrovirus strain (ITA/2008/PR3147) was identified (Figure, panel B). The patient was a child, 14 months of age, who was hospitalized for 4 days with severe gastroenteritis during November 2008. Sequence analysis of the small ORF2 fragment targeted by the MLB1-specific primers (*2*) revealed that the MLB1-like astrovirus was highly similar (99.7% nt identity) to strain ITA/2007/PR326.

These unusual astroviruses have been identified in fecal samples of persons in Australia and in countries of Asia, Africa, and North America (*2*–*6*,*10*). In this study, MLB1-like astroviruses were identified in children with severe gastroenteritis, thus extending information on geographic diffusion of these viruses to the continent of Europe. Yet, the epidemiologic information on these viruses is limited and scattered. Structured virologic and serologic studies and effective diagnostic tools are necessary to assess more properly the epidemiology and role of these viruses in humans.
